# Diagnostic Ureterorenoscopy Is Associated with Increased Intravesical Recurrence following Radical Nephroureterectomy in Upper Tract Urothelial Carcinoma

**DOI:** 10.1371/journal.pone.0139976

**Published:** 2015-11-10

**Authors:** Hyun Hwan Sung, Hwang Gyun Jeon, Deok Hyun Han, Byong Chang Jeong, Seong Il Seo, Hyun Moo Lee, Han-Yong Choi, Seong Soo Jeon

**Affiliations:** Department of Urology, Samsung Medical Center, Samsung Biomedical Research Institute, Sungkyunkwan University School of Medicine, Seoul, Korea; Sun Yat-sen University, CHINA

## Abstract

Diagnostic ureterorenoscopy is powerful tool to confirm upper tract urothelial cancer (UTUC). However, URS and associated manipulation may be related to the risk of intravesical recurrence (IVR) following radical nephroureterectomy (RNU). We aimed to investigate whether preoperative ureterorenoscopy would increase IVR after RNU in patients with UTUC. We performed a retrospective analysis of 630 patients who had RNU with bladder cuff excision due to UTUC. Diagnostic URS was performed in 282 patients (44.7%). Patients were divided into two groups according to the URS. Survival analysis and multivariate Cox regression model were performed to address risk factors for the IVR. The interval from URS to RNU was measured. During URS, manipulation such as biopsy and resection was determined. The median age was 64 (IQR 56–72) years with follow-up duration of 34.3 (15.7–64.9) months. Median time from URS to RNU was 16 (0–38) days. The IVR developed in 42.5% (*n* = 268) patients at 8.2 (4.9–14.7) months. The five-year IVR-free survival rate was 42.6 ± 8.0% and 63.6 ± 6.9% in patients with and without preoperative URS, respectively (*P* < 0.001). In multivariate analysis, previous history of bladder tumour, extravesical excision of distal ureter, multifocal tumour, and URS (HR, 95% CI; 1.558, 1.204–2.016, *P* = 0.001) were independent predictors for higher IVR. The IVR rate in patients without manipulation during URS was not different to those with manipulation (*P* = 0.658). The duration from URS to RNU was not associated with IVR (*P* = 0.799). Diagnostic URS for UTUC increased IVR rate after RNU. However, the lessening of interval from URS to radical surgery or URS without any manipulation could not reduce the IVR rate.

## Introduction

Upper tract urothelial cancer (UTUC) is a rare and aggressive malignant disease, accounting for about 5% of urothelial carcinoma [1, 2]. Radical nephroureterectomy (RNU) with partial bladder cuff excision is the gold standard treatment for localized UTUC [3]. Compared to bladder cancer, the prognosis for patients with UTUC remains relatively poor. The natural history of UTUC differs from that of bladder cancer, and about 60% of UTUCs are invasive at diagnosis compared with only 15–25% of bladder tumors [4, 5]. Approximately 20 to 50% of patients with UTUC experience recurrence in the bladder after standard treatment with RNU [6]. Intravesical recurrence (IVR) following RNU may be related to the risk of disease progression and contralateral involvement of UTUC, as well as the overall cost of treatment and poor quality of life resulting from intense cystoscopic surveillance and transurethral resection of bladder tumors [7].

There have been several studies aimed at identifying the risk factors of IVR based on perioperative clinicopathologic features [8–11]. Recently, the UTUC collaboration group suggested a nomogram that predicts IVR after RNU with reasonable accuracy [11]. The full model of this nomogram includes age, gender, previous bladder cancer, tumor location, stage, concomitant CIS, lymph node (LN) status, and laparoscopic approach as risk factors for IVR.

In the patients with uncertain and small upper urinary tract tumors, preoperative diagnostic ureterorenoscopy (URS) has been the most powerful diagnostic tool to confirm UTUC. However, the URS procedure and associated manipulation may increase the risk of intraluminal tumor seeding and IVR. There have been limited reports of the outcome of bladder tumor recurrence, and the impact of URS on IVR following RNU has remained controversial [9, 11, 12]. One recent study found that URS was associated with an increased incidence of intravesical tumor recurrence [9]. However, the authors noted that a major flaw of their study was the lack of flexible URS use for renal pelvis tumors. In the current study, we aimed to investigate whether diagnostic URS would increase the IVR and be an independent predictor for the IVR following RNU in patients with UTUC.

## Materials and Methods

This study was approved by the Institutional Review Board of Samsung Medical Center at Sungkyunkwan University School of Medicine (IRB Number: 2013-10-121). We could not get consent from the patients because this study was retrospectively designed.

### Patients

From 1994 to 2013, 683 patients underwent RNU with bladder cuff excision due to presumed UTUC. Among these patients, 53 were excluded for the following reasons: the presence of other malignancies or benign diseases in the final pathologic reports; bilateral UTUC; prior cystectomy due to invasive bladder cancer; and other miscellaneous causes. The medical records of the remaining 630 patients were retrospectively reviewed for this study.

Perioperative clinicopathologic data including sex, age, American Society of Anesthesiologists (ASA) score, type of bladder cuff excision, prior bladder tumor history, location of tumor, tumor stage, grade, size, carcinoma in situ (CIS), lymphovascular invasion (LVI), margin status, adjuvant chemotherapy, IVR, and disease progression were obtained from chart review. To analyse effect of the time period of operation on IVR, patients were divided into the quintile according to serial case number. Tumor stage was reassessed according to the 2002 American Joint Committee of Cancer tumor-node-metastasis (TNM) classification. The WHO–International Society of Urologic Pathology consensus classification was used to evaluate tumor grade. Clinical and pathological data were compared between two groups according to preoperative URS. Ureterorenoscopy was performed in case of uncertain and small upper urinary tract tumors, or surgeon’s preference to confirm pathology prior to radical surgery. Depending on the location of the tumor, rigid and/or flexible URS was performed. We analysed whether manipulation such as tumor biopsy was performed or not during ureterorenoscopic procedure. The time from diagnostic URS to RNU was determined to investigate the effect of concurrent or delayed radical surgery on IVR. Immediate intravesical instillation of chemotherapeutic agents was not carried out at our institution just after the diagnostic URS. The IVR rate following RNU was evaluated and compared between the groups. The IVR was assessed by a regular cystoscopic exam or computed tomography (CT) followed by pathological confirmation.

An additional incision was created for bladder cuff excision on each side of low abdomen regardless of the laparoscopic or open method. Method of cuff excision is classified into intravesical vs. extravesical approach. Endoscopic management of distal ureter has never been done in our institution.

### Patient follow-up

All patients were followed every 3–4 months for the first year after surgery, every six months from the second to fifth years, and annually thereafter with cystoscopic examination, urine cytology, and routine check-ups that included history, physical exam, and blood tests at each follow-up visit. Imaging evaluations using CT or magnetic resonance imaging (MRI) were performed every six months for the first five years and then annually thereafter. Chest CT and bone scans were evaluated if clinically indicated.

### Statistical analysis

All continuous variables are shown as median value and interquartile range (IQR). Survival rates are shown with 95% confidence intervals (CI). Statistical significance was assessed based on a two-sided *P*-value less than 0.05.

Student's t-test was used to compare continuous variables between the two groups, and the distributions of categorical variables were compared using Pearson’s Chi-square test. The Kaplan–Meier method was used to calculate survival outcomes, and the log-rank test was used to assess differences.

Multivariate analysis was performed using a Cox’s proportional-hazard method. To derive a final model of the variables with a significant independent relationship with survival, variables with *P* value less than 0.20 in the univariate analysis were enrolled into multivariate analysis. All statistical analysis was performed with IBM SPSS Statistics Version 22.0 (IBM, Armonk, New York, USA).

## Results

### Baseline characteristics

The median patient age was 64 (IQR 56–72) years. Median follow-up duration was 34.3 (15.7–64.9) months. Preoperative URS was performed in 44.7% (*n* = 282) of patients. Recent patient had more URS, and more than half underwent URS in the last three quintiles (Table 1). The median time from URS to RNU was 16 (0–38) days. Of these, 72 patients performed diagnostic URS and following RNU at the same day. During URS, 92.6% (*n* = 261) were performed with manipulation. Prior or concurrent bladder tumor was found in 19.5% (*n* = 123) of patients. There was no significant difference between the two groups with regard to age, gender, location of tumor, multifocality, grade, LN status and adjuvant chemotherapy (Table 1). However, non-URS group had longer follow-up duration, more transvesical resection in the distal ureter management, more advanced pathologic tumor stage, larger tumor, greater history of previous bladder tumor, more positive margin and fewer multifocal tumors than the preoperative URS group (*P* < 0.05; Table 1).

**Table 1:** Baseline characteristics of all patients, and clinicopathologic features according to preoperative ureterorenoscopy.

	All patients (*n* = 630)	PreOP URS (-) (*n* = 348)	PreOP URS (+) (*n* = 282)	*P* value
Sex, male, % (n)	73.8 (465)	72.7 (253)	75.2 (212)	0.482
Age, yr, median (IQR)	64 (56–72)	65 (55–71)	64 (57–72)	0.752
FU duration, months, median (IQR)	34.3 (15.7–64.9)	39.3 (16.1–80.1)	30.1 (15.2–54.1)	<0.001
Case number (quintile), % (n)				<0.001
1~126		84.9 (107)	15.1 (19)	
127~252		72.2 (91)	27.8 (35)	
253~378		43.7 (55)	56.3 (71)	
379~504		37.3 (47)	62.7 (79)	
505~630		38.1 (48)	61.9 (78)	
Laterality, Rt, % (n)	46.3 (292)	45.1 (157)	47.9 (135)	0.490
ASA score, II or greater, % (n)	68.6 (432)	67.5 (235)	69.9 (197)	0.531
Ureter involved, % (n)	50.2 (316)	49.1 (171)	51.4 (145)	0.569
Multifocal tumor, % (n)	26.7 (168)	25.0 (87)	28.7 (81)	0.293
Previous bladder tumor, % (n)	19.5 (123)	22.7 (79)	15.6 (44)	0.025
Surgical approach, % (n)				<0.001
Open	62.2 (392)	72.4 (252)	49.6 (140)	
Laparoscopic	37.8 (238)	27.6 (96)	50.4 (142)	
Bladder cuffing, transvesical, % (n)	57.3 (361)	63.8 (222)	49.3 (139)	<0.001
Tumor grade, III, % (n)	44.9 (277)	44.5 (153)	45.4 (124)	0.815
Tumor stage				<0.001
Ta	17.3 (109)	14.4 (50)	20.9 (59)	
T1	24.3 (153)	19.8 (69)	29.8 (84)	
T2	16.3 (103)	15.8 (55)	17.0 (48)	
T3 and T4	42.1 (265)	50.0 (174)	32.3 (91)	
LN status, positive, % (n)	8.9 (56)	10.3 (36)	7.1 (20)	0.154
CIS, positive, % (n)	10.5 (66)	8.6 (30)	12.8 (36)	0.094
Tumor size, cm, median (IQR)	3.5 (2.3–5.0)	4.0 (3.0–5.5)	3.0 (1.8–4.0)	<0.001
Margin, positive, % (n)	4.9 (31)	6.6 (23)	2.8 (8)	0.030
LVI, positive, % (n)	17.1 (108)	17.8 (62)	16.3 (46)	0.618
Adjuvant chemotherapy, % (n)	20.3(128)	21.3 (74)	19.1 (54)	0.512

ASA, American Society of Anesthesiologists; CIS, carcinoma in situ; FU, follow-up; HR, hazard ratio; CI, confidence interval; IQR, interquartile range; LN, lymph node; LVI, lymphovascular invasion; URS, ureterorenoscopy.

### Intravesical recurrence following radical nephroureterectomy

**Fig 1:**
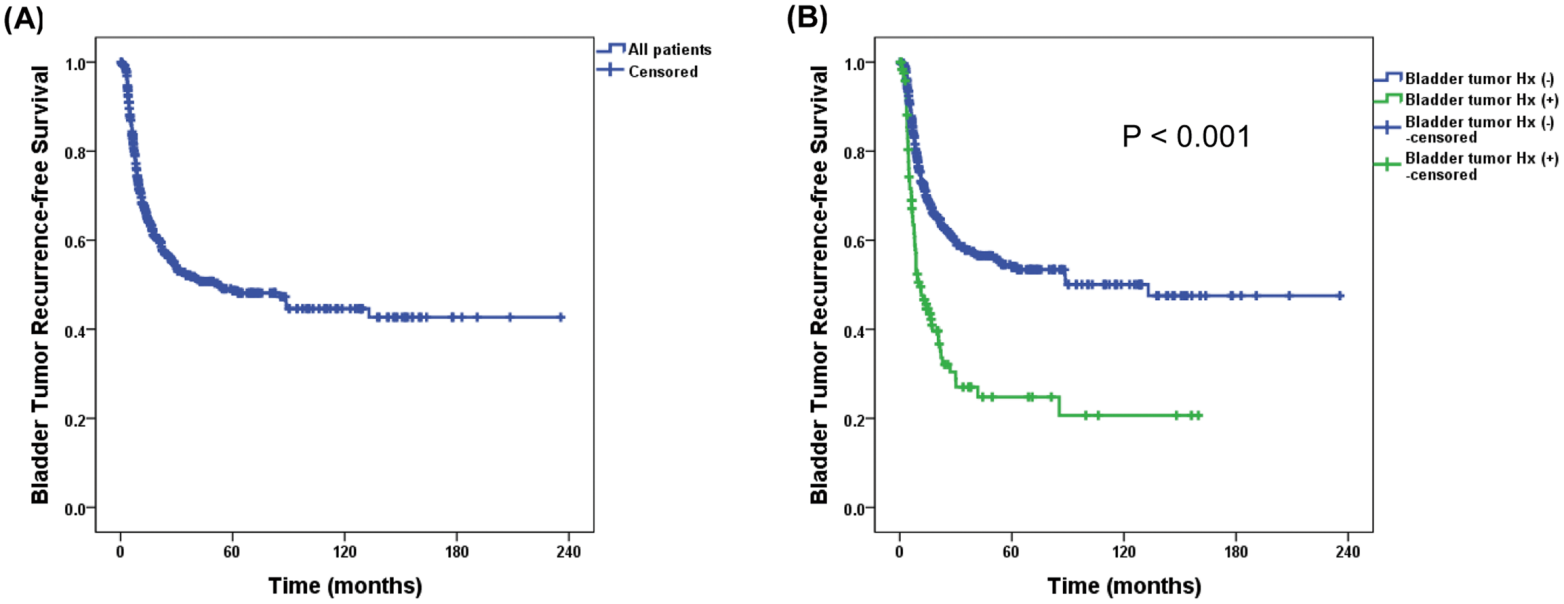
Survival analysis of intravesical recurrence in all patients (A), and according to previous bladder tumor history (B) following radical nephroureterectomy.

**Fig 2:**
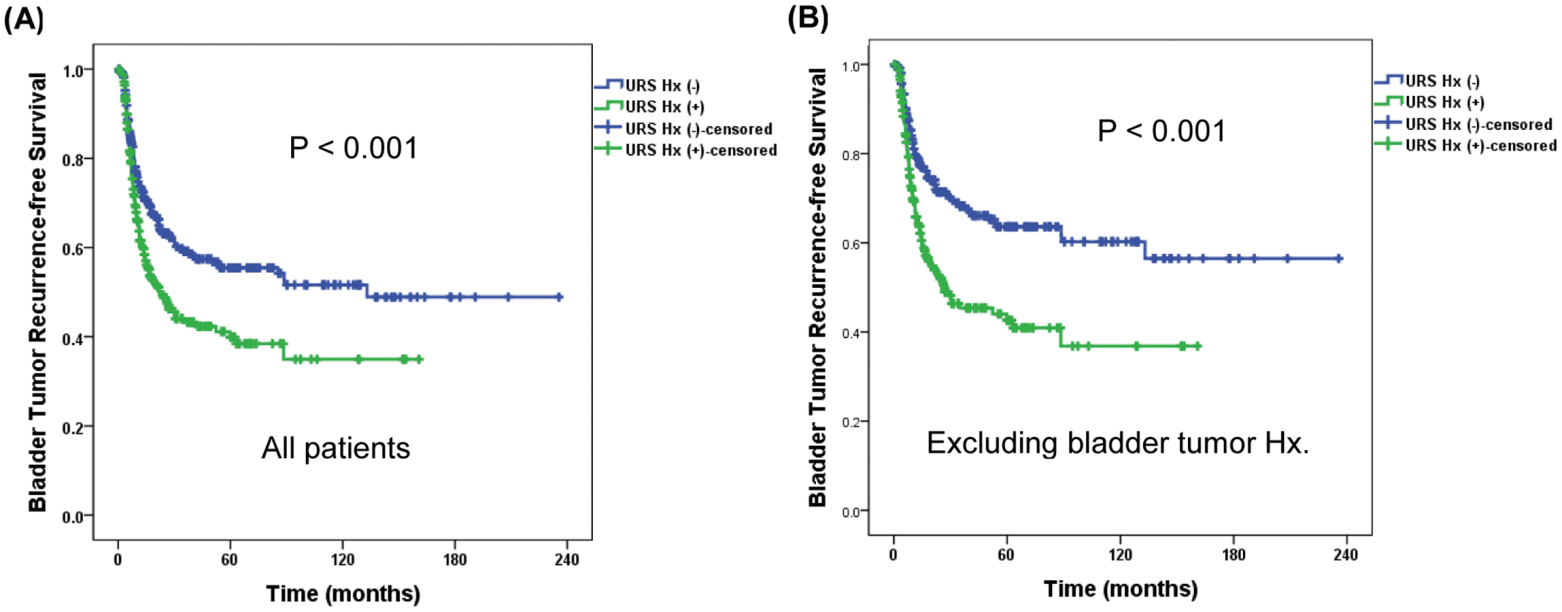
Survival analysis of intravesical recurrence according to preoperative ureterorenoscopy in all patients (A, *n* = 630) and excluding prior bladder tumor history (B, *n* = 507) following radical nephroureterectomy.

During the follow-up, the IVR developed in 42.5% (*n* = 268) of all patients at a median of 8.2 (4.9–14.7) months after RNU. A few recurrences were observed even 5-years after the RNU (*n* = 6, 2.2%), although most recurrences occurred within three years after the RNU (253/268 cases, 94.4%, Fig 1A). Patients with bladder tumor history had more IVR (*P* < 0.001; Fig 1B). Time to IVR in patients with prior bladder tumor was earlier than those without bladder tumor (6.6 (IQR 4.2–11.3) vs. 8.9 (5.6–16.1) months, *P* = 0.048). The group with preoperative URS had significantly higher IVR rate irrespective of prior bladder tumor history (*P* < 0.001; Fig 2A and 2B). Excluding the prior bladder tumor history, the five-year IVR-free survival rate was 42.6 ± 8.0% and 63.6 ± 6.9% in patients with and without preoperative URS, respectively (*P* < 0.001, Fig 2B).

### Analysis for predicting intravesical recurrence following radical nephrouterectomy

In terms of the type of bladder cuff excision, extravesical resection of the distal ureter was associated with high IVR (*P* < 0.001). Patients with multifocal, positive margin, prior bladder tumor history, and preoperative URS experienced more IVR after RNU (*P* < 0.05, Table 2). In the multivariate analysis, previous history of bladder tumor (HR, 95% CI; 2.535, 1.903–3.376, *P* < 0.001), extravesical excision of the distal ureter (1.411, 1.075–1.852, *P* < 0.001), multifocal tumor (1.398, 1.055–1.853, *P* = 0.020), and diagnostic URS (1.558, 1.204–2.016, *P* = 0.001) still remained significant independent risk factors to predict the IVR following the RNU (Table 2). Patients with positive resection margin have a trend of higher IVR (*P* = 0.054). Time period of operation was not a risk factor in the multivariate analysis.

**Table 2:** Risk factors for predicting intravesical recurrence following radical nephroureterectomy for upper tract urothelial cancer in 630 patients.

	Univariate Analysis		Multivariate Analysis	
	HR (95% CI)	*P* value	HR (95% CI)	*P* value
Sex, female	0.919 (0.692–1.221)	0.561		
Age, continuous	1.007 (0.995–1.018)	0.264		
Case number (quintile)		0.008		0.099
1~126	Referent		Referent	
127~252	1.432 (0.967–2.121)	0.073	1.152 (0.770–1.723)	0.490
253~378	2.042 (1.389–3.001)	<0.001	1.457 (0.953–2.230)	0.083
379~504	1.629 (1.081–2.453)	0.020	0.942 (0.591–1.501)	0.801
505~630	1.586 (1.009–2.494)	0.046	0.936 (0.571–1.534)	0.793
ASA score, II or greater	1.216 (0.936–1.581)	0.143	1.067 (0.815–1.396)	0.718
Ureter involvement	1.158 (0.911–1.472)	0.232		
Multifocal tumor	1.397 (1.076–1.814)	0.012	1.398 (1.055–1.853)	0.020
Previous bladder tumor history	2.369 (1.813–3.095)	<0.001	2.535 (1.903–3.376)	<0.001
Surgical approach, laparoscopic	1.087 (0.847–1.394)	0.512		
Cuffing type, extravesical	1.568 (1.229–1.999)	<0.001	1.411 (1.075–1.852)	0.013
Tumor grade, III	0.984 (0.770–1.257)	0.897		
Tumor stage		0.881		
Ta	Referent			
T1	1.066 (0.740–1.534)	0.733		
T2	1.094 (0.737–1.623)	0.656		
T3 or 4	0.967 (0.691–1.354)	0.847		
LN, positive	0.641 (0.374–1.099)	0.106	0.558 (0.322–0.967)	0.038
CIS, positive	1.304 (0.914–1.861)	0.143	0.827 (0.566–1.209)	0.328
Size, continuous	1.027 (0.979–1.078)	0.277		
Margin, positive	1.994 (1.232–3.228)	0.005	1.646 (0.992–2.732)	0.054
LVI, positive	0.912 (0.647–1.286)	0.598		
Adjuvant chemotherapy	0.860 (0.633–1.166)	0.331		
Ureterorenoscopy	1.550 (1.218–1.972)	<0.001	1.558 (1.204–2.016)	0.001

ASA, American Society of Anesthesiologists; CIS, carcinoma in situ; HR, hazard ratio; CI, confidence interval; IQR, interquartile range; LN, lymph node; LVI, lymphovascular invasion; URS, ureterorenoscopy.

### Effect of manipulation during ureterorenoscopy on intravesical recurrence

**Fig 3:**
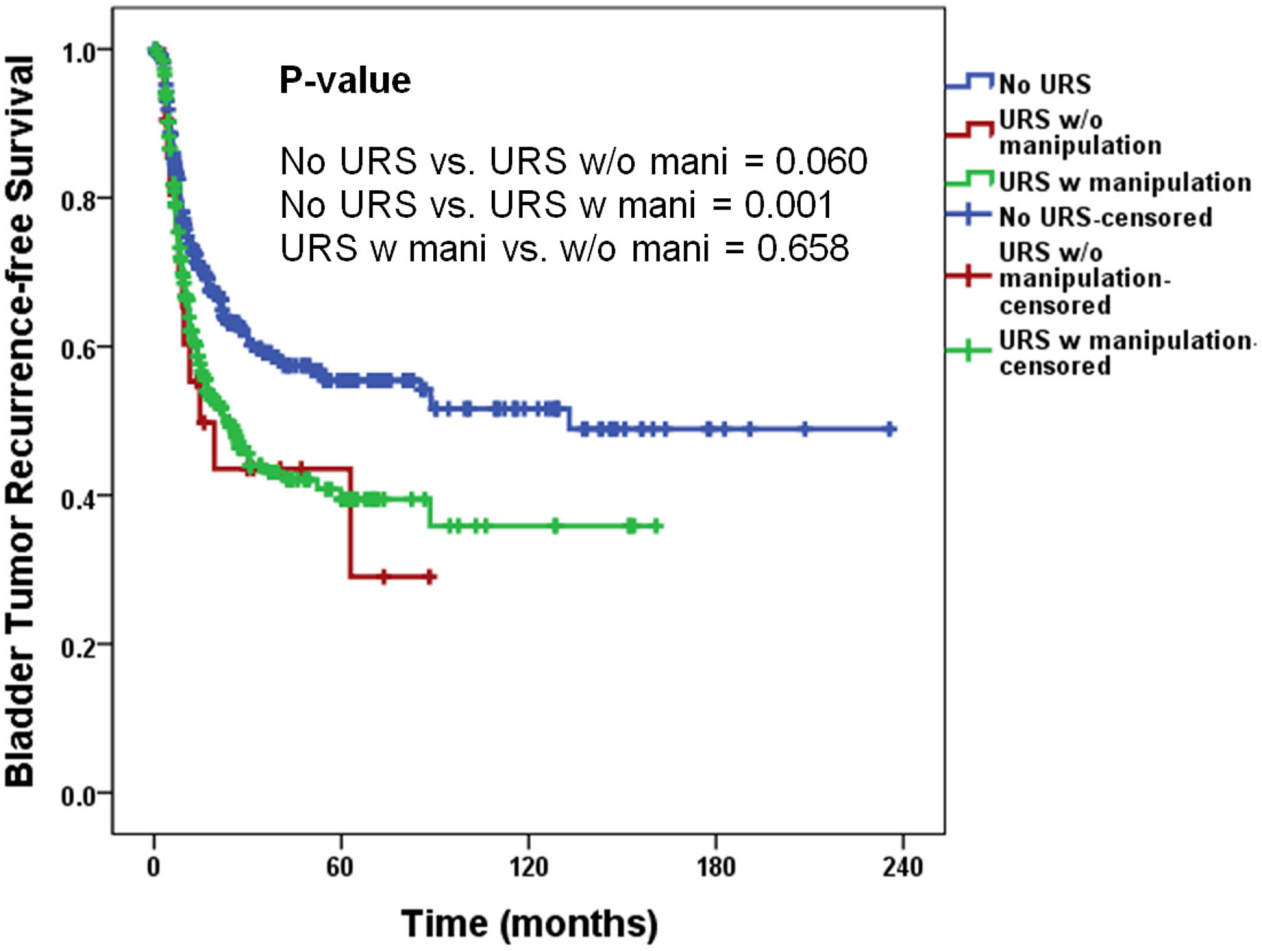
Survival analysis of intravesical recurrence according to manipulation. mani, manipulation; URS, ureterorenoscopy; w, with; w/o, without.

During diagnostic URS, 7.4% (*n* = 21) patients did not take any manipulation such as tumor biopsy and balloon dilation. The IVR rate in patients without manipulation during URS was not different to those with manipulation (*P* = 0.658), and higher than those without URS although there was a statistically marginal significance (*P* = 0.060, Fig 3).

### Effect of the duration between ureterorenoscopy and radical nephroureterectomy on intravesical recurrence

**Fig 4:**
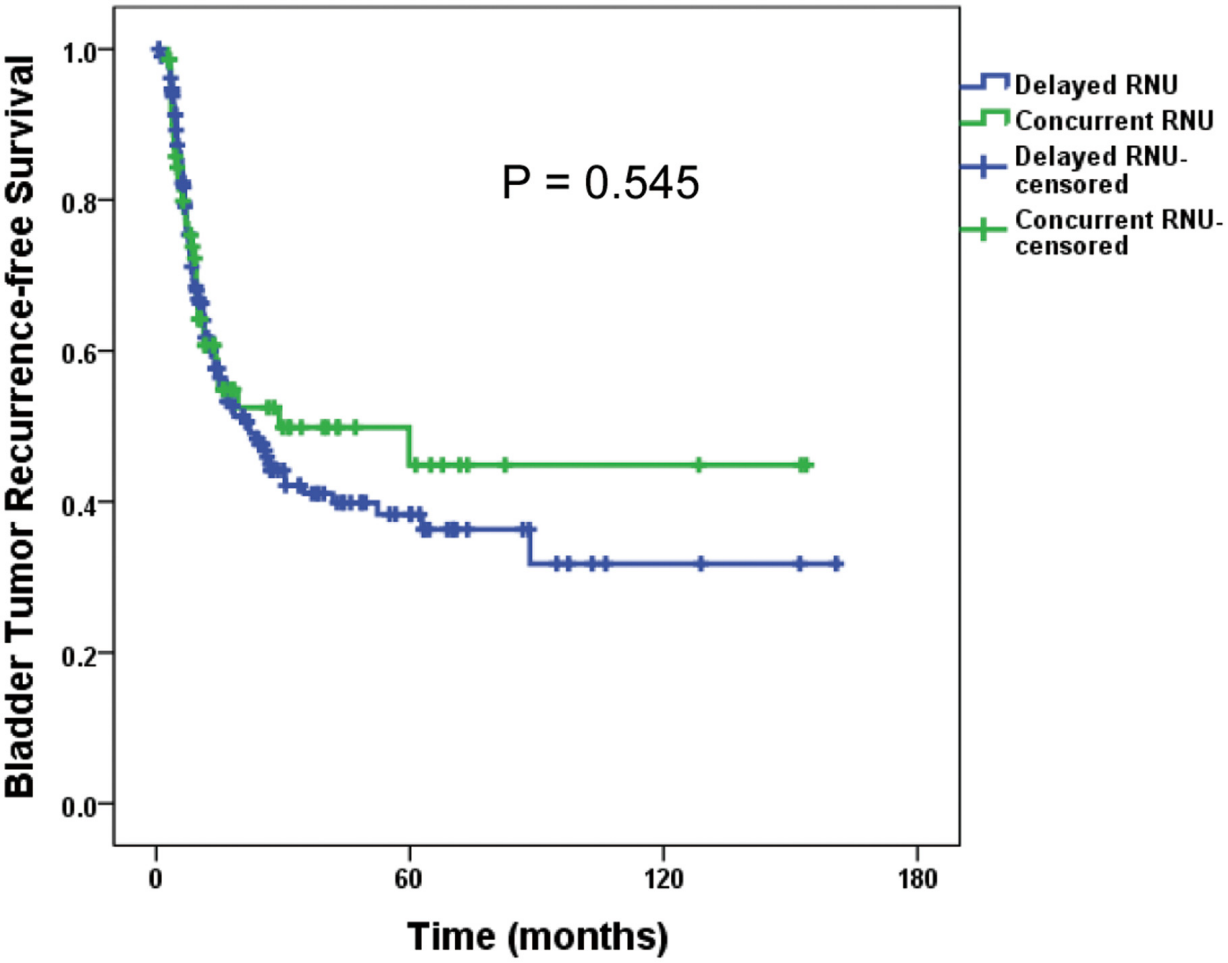
Survival analysis of intravesical recurrence between concurrent ureterorenoscopy with radical nephroureterectomy group (*n* = 72) versus preceding ureterorenoscopy and delayed radical nephroureterectomy group (*n* = 210).

In the patients who had preoperative URS, the duration from URS to RNU was not associated with IVR (HR, 95% CI; 1.000, 0.998–1.002, *P* = 0.799). We also compared the incidence of bladder tumor recurrence between concurrent RNU with URS (operations at the same day, *n* = 72) and delayed RNU after URS (operations at separate day, *n* = 210). The IVR rate of the delayed RNU group did not differ from that of the concurrent RNU group (*P* = 0.545, Fig 4).

## Discussion

Several theories have been suggested to explain the IVR following the RNU, including intraluminal tumor seeding, intraepithelial cancer migration, and urinary tract cancerization [9, 13]. These theories have also been applied to the IVR following the preoperative URS. However, the effects of preoperative diagnostic URS on the IVR following the RNU in patients with UTUC have not been determined [9, 11, 12]. Here, we found that the diagnostic URS was significantly associated with increased IVR, irrespective of the history of bladder tumor. In addition, history of bladder tumor, extravesical cuff excision, and multifocal tumor were significant risk factors to predict the IVR. Although more recent patients have undergone more preoperative URS, the time period of operation was not related to higher IVR.

The oncologic effect and the IVR of prolonged RNU are not well understood in patients who undergo the diagnostic URS. Recently, Nison et al. reported the oncologic impact of a delay between diagnosis and RNU [14]. They showed that the implementation of diagnostic URS increases the time to RNU without significantly altering cancer-specific, recurrence-free or metastasis-free survival, even for muscle-invasive lesions. However, they did not show the effect of a delay between diagnosis and RNU on bladder recurrence. Because the patients with delayed RNU could be exposed to risk for a longer period of intraluminal tumor seeding after biopsy or manipulation, the impact of delayed RNU should be considered. Thus, we also evaluated the relationship between the IVR and time lag of radical surgery in patients who underwent URS. We did not find any differences between the concurrent and delayed RNU groups with respect to the incidence of IVR or time to bladder recurrence, with a median time from URS to RNU of 16 (0–38) days.

In our study, survival analyses of bladder tumor recurrence were performed after excluding previous bladder tumor history, which is a well-known prognostic factor of the IVR [11, 15–17]. In the multivariate analysis, the bladder tumor history remained the most important risk factor. Multifocal tumor was also a significant risk factor associated with the IVR, although the prognostic impact of multifocality of UTUC remains poorly understood [8, 12, 18, 19]. Chromecki et al. reported that the tumor multifocality is an independent prognostic factor of disease progression and cancer-specific mortality [18]. However, they did not mention the impact on bladder tumor recurrence. A recently developed nomogram to predict the IVR also did not include the multiplicity of tumors [11]. On the other hand, some reports found that the multifocal tumor was a risk factor [8, 9, 19]. They proposed that previous bladder cancer history and multifocal tumor may be associated with field urinary tract cancerization and intraepithelial cancer migration [9].

The impact of distal ureter management on the IVR is still under debate, although different techniques have comparable oncologic outcomes, including disease progression and cancer specific mortality [11, 19–22]. Different outcomes of the impact of distal ureter management have been reported, even by same research group. The UTUC collaboration group first showed that there was no difference between the transvesical and extravesical approaches in bladder tumor recurrence, but they included the extravesical method as a risk factor in the recent nomogram predicting the IVR [11, 22]. Our study showed that the extravesical approach was a significant independent predictor for the IVR. This result could be limited by the lack of the use of endoscopic distal ureter management method at our institution. A more comprehensive evaluation of the IVR is therefore needed to verify the impact of distal ureter management following RNU for the UTUC.

Patients who underwent laparoscopic RNU did not show an association with bladder tumor recurrence in our study. In the beginning period, laparoscopic RNU has been suggested as a risk factor for oncologic outcome and the IVR. Initial data resulted that the high gas pressure during the procedure and a longer operative time owing to lack of operator experience might promote tumor cell dissemination within the urinary tract [15, 23]. Recently, there have been some evidences supporting that laparoscopic RNU as a minimally invasive surgery alternative to open RNU based on no differences in oncologic outcomes [10, 24–27].

There have been several trials to reduce IVR after RNU. Recent reports have suggested that immediate intravesical chemotherapy following RNU reduces the risk of bladder tumor [6, 7, 28]. Intravesical mitomycin-C instillation following RNU reduced the probability of IVR with an absolute risk reduction of 11%, although the results of the modified intent-to-treat analysis were not statistically significant (*P* = 0.055) [7]. Based on these results, a probability nomogram predicting IVR was developed to improve the clinical decision-making process regarding postoperative intravesical instillations of MMC after RNU [11]. In another randomized phase II study, a single intravesical instillation of pirarubicin appeared to reduce bladder recurrence [28]. In order to reduce the IVR, several methods such as early bladder cuff excision and intravesical chemotherapy after diagnostic URS need to be considered. In patients who have already undergone URS, we had expected that delayed radical surgery or manipulation during URS could increase the IVR, probably due to prolonged or increased potential of tumor seeding. However, our data revealed that the radical surgery with URS at the same session or URS without manipulation (just look) did not reduce the IVR rate.

We note that our study had several limitations, first and foremost being its retrospective study design. The URS group and the non-URSgroup differ significantly from each other. This is clearly the major limitation of this paper. Despite controlling for all appropriate variables, there could be other unmeasured confounders that are biasing the findings here. Second, several surgeons were involved in the study. More patients who underwent laparoscopic surgery also had more preoperative diagnostic URS and extravesical approach in distal ureter management, although all surgeons at our institution followed similar oncologic surgical principles. Third, there was no definitive standard criteria for performing diagnostic URS before the RNU. Preoperative URS has generally been undertaken in patients with uncertain diagnosis and willing to obtain pathologic proof. Thus, patients who underwent URS appeared to have small-sized and early-stage tumors. This study needs to be validated with a prospective design.

## Conclusions

The diagnostic URS for UTUC was associated with the increased IVR rate following RNU. Prior bladder tumor history, multifocal UTUC, and extravesical cuff excision were also predictors for the IVR. However, the lessening of interval from URS to radical surgery or URS without any manipulation could not reduce the IVR rate. Further prospective studies are needed to verify this result, and a protocol to prevent the IVR after the diagnostic URS and RNU should be developed.
